# Omega‐3 fatty acid supplements and recurrent miscarriage: A perspective on potential mechanisms and clinical evidence

**DOI:** 10.1002/fsn3.3464

**Published:** 2023-06-01

**Authors:** Fangxiang Mu, Huyan Huo, Mei Wang, Fang Wang

**Affiliations:** ^1^ Department of Reproductive Medicine Lanzhou University Second Hospital Lanzhou China

**Keywords:** endocrine disorder, immunity, omega‐3 fatty acid, oxidative stress, recurrent miscarriage

## Abstract

Recurrent miscarriage (RM) affects approximately 1%–5% of couples worldwide. Due to its complicated etiologies, the treatments for RM also vary greatly, including surgery for anatomic factors such as septate uterus and uterine adhesions, thyroid modulation drugs for hyperthyroidism and hypothyroidism, and aspirin and low molecular weight heparin for antiphospholipid syndrome. However, these treatment modalities are still insufficient to solve RM. Omega‐3 fatty acids are reported to modulate the dysregulation of immune cells, oxidative stress, endocrine disorders, inflammation, etc., which are closely associated with the pathogenesis of RM. However, there is a lack of a systematic description of the involvement of omega‐3 fatty acids in treating RM, and the underlying mechanisms are also not clear. In this review, we sought to determine the potential mechanisms that are highly associated with the pathogenesis of RM and the regulation of omega‐3 fatty acids on these mechanisms. In addition, we also highlighted the direct and indirect clinical evidence of omega‐3 fatty acid supplements to treat RM, which might encourage the application of omega‐3 fatty acids to treat RM, thus improving pregnancy outcomes.

## Graphical abstract

We highlighted the direct and indirect clinical evidence of omega‐3 fatty acid supplements to treat RM, which might encourage the application of omega‐3 fatty acids to treat recurrent miscarriage, thus improving their pregnancy outcomes.

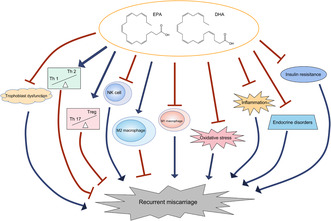

## 1 INTRODUCTION

Recurrent miscarriage (RM) is a critical disorder of pregnancy that affects approximately 1%–5% of couples worldwide (Dimitriadis et al., 2020). The Royal College of Obstetricians and Gynaecologists has defined RM as three or more spontaneous miscarriages, while the American Society of Reproductive Medicine and the European Society of Human Reproduction and Embryology both defined RM as two or more failures of pregnancy (ESHRE Guideline Group on RPL, 2018; Green & O'Donoghue, 2019; Practice Committee of the American Society for Reproductive Medicine, 2012). The potential etiologies of RM are quite complex and include male factors (such as male partner's sperm quantity, sperm quality, and genetic mutations), and genetic abnormality, anatomic issues, and immune disorders (Deshmukh & Way, 2019; Quenby et al., 2021; Yu & Bao, 2022). Therefore, the treatment of RM also varies greatly and includes surgery, immunotherapies, anticoagulants, endocrine therapies, etc. (Deng et al., 2022; Uthman et al., 2019; Wong et al., 2014). However, current management is still insufficient to solve RM.

Omega‐3 fatty acids are a group of polyunsaturated fatty acids that mainly refer to α‐linolenic acid (ALA), eicosapentaenoic acid (EPA), and docosahexaenoic acid (DHA) in terms of human physiology (Bradberry & Hilleman, 2013; Brinton & Mason, 2017). ALA, merely obtained from the diet, can be metabolized into EPA and DHA in humans, but the metabolized EPA and DHA are insufficient due to dietary habits (Bradberry & Hilleman, 2013). Therefore, it is important to directly take in EPA and DHA from dietary sources, such as fish oil and other sea foods (DiNicolantonio & O'Keefe, 2020). In terms of RM, there is some evidence suggesting the potential involvement of omega‐3 fatty acids in treating or preventing RM (Carta et al., 2005; Lazzarin et al., 2009; Rossi & Costa, 1993). In addition, omega‐3 fatty acids possess anti‐inflammatory, immune‐modulatory, and endocrine‐modulatory effects, which could possibly affect RM (Di Bari et al., 2017; Dimitriadis et al., 2020; Rees et al., 2022; Singh, 2020; Tajuddin et al., 2016). However, there is no general consensus on the benefits of omega‐3 fatty acid supplements in treating RM as well as the fundamental mechanism of omega‐3 fatty acids in disputing RM. This study aimed to review the potential involvement of omega‐3 fatty acids in RM.

## 2 CURRENT OPINIONS ON RM

### 2.1 Epidemiology, risk factors, and treatment

RM is known for hampering the productivity of humans, and it also places huge stress on patients. It has been reported that couples, especially women with RM, bear huge psychological stress (Banno et al., 2020). The average prevalence of women with one previous miscarriage is 10.8%, that of women with two miscarriages is 1.9%, and that of women with three or more miscarriages is 0.7% in the West (Quenby et al., 2021). Taking the definition of RM as two or more previous miscarriages, the prevalence of miscarriage is obtained to be 2.6% in the West. However, the incidence of RM varies among regions and is reported to be 1%–5% (Dimitriadis et al., 2020; Quenby et al., 2021). Even worse, studies conducted in developing countries are lacking; thus, the actual data might vary. In the past few decades, several risk factors for RM have been identified. For instance, previous times of miscarriage is a critical risk factor for RM; individuals who experience six or more miscarriages face a nearly 60% risk of further miscarriage (Coomarasamy et al., 2020). Age is also a well‐recognized risk factor; individuals with an age range of 20–29 years have a lower risk of RM, while those with an age below 20 or above 30 have a higher risk of RM (Dimitriadis et al., 2020; Quenby et al., 2021). Other typical risk factors include obesity [body mass index (BMI) ≥ 30], stress, smoking, high caffeine or alcohol intake during the first trimester, thyroid dysfunction, polycystic ovary syndrome, antiphospholipid syndrome, etc. (Chakraborty et al., 2013; Dong et al., 2020; Sugiura‐Ogasawara, 2015; Uthman et al., 2019).

The treatments of RM rely on the etiologies of RM, which vary greatly and mainly include anatomic factors, endocrine factors, immune factors, etc. Regarding the common anatomic factors of RM, such as septate uterus and uterine adhesions, they can be treated with surgery. For instance, a meta‐analysis suggests that RM caused by a septate uterus can be managed by hysteroscopic metroplasty, although there exists a potential risk of postoperative complications (Valle & Ekpo, 2013). For patients with RM induced by uterine adhesions, hysteroscopic adhesiolysis is the common surgical management, combined with antiadhesion barriers if needed (Vitale et al., 2022).

Hyperthyroidism, hypothyroidism, and polycystic ovary syndrome are common endocrine etiologies of RM (Amrane & McConnell, 2019). The treatment of RM due to hyperthyroidism and hypothyroidism mainly includes antithyroid drugs (such as propylthiouracil and methimazole) and levothyroxine (Negro & Stagnaro‐Green, 2014). However, the dose of these agents should be carefully adjusted. In terms of polycystic ovary syndrome, the currently recommended management includes improvement of dietary intake, more physical activities, and weight control (Akre et al., 2022). In addition, metformin is also recommended to assist in weight management and insulin resistance (Zhao & He, 2022).

Antiphospholipid syndrome is a well‐recognized immune etiology of RM that affects approximately 15%–30% of patients with RM (Schreiber et al., 2018). Currently, aspirin (75–100 mg/day) or low molecular weight heparin (dosage not mentioned) is recommended to manage antiphospholipid syndrome in patients with RM (ESHRE Guideline Group on RPL et al., 2018). Recently, several meta‐analyses revealed that the combination of aspirin and low molecular weight heparin may improve the live birth rate compared with patients that received aspirin only (Hamulyak et al., 2020; Li et al., 2020; Liu et al., 2020).

However, the etiologies of approximately 50% of RM are still unclear (Tur‐Torres et al., 2017). Generally, several factors, including trophoblast dysfunction and relevant signaling pathways, genetic polymorphism, and immune dysfunction, may contribute to the pathogenesis of RM. Unfortunately, the treatment of patients with RM of unknown etiology remains unsatisfactory.

### 2.2 Underlying mechanisms

#### 2.2.1 Trophoblast dysfunction and relevant signaling pathways

**FIGURE 1:**
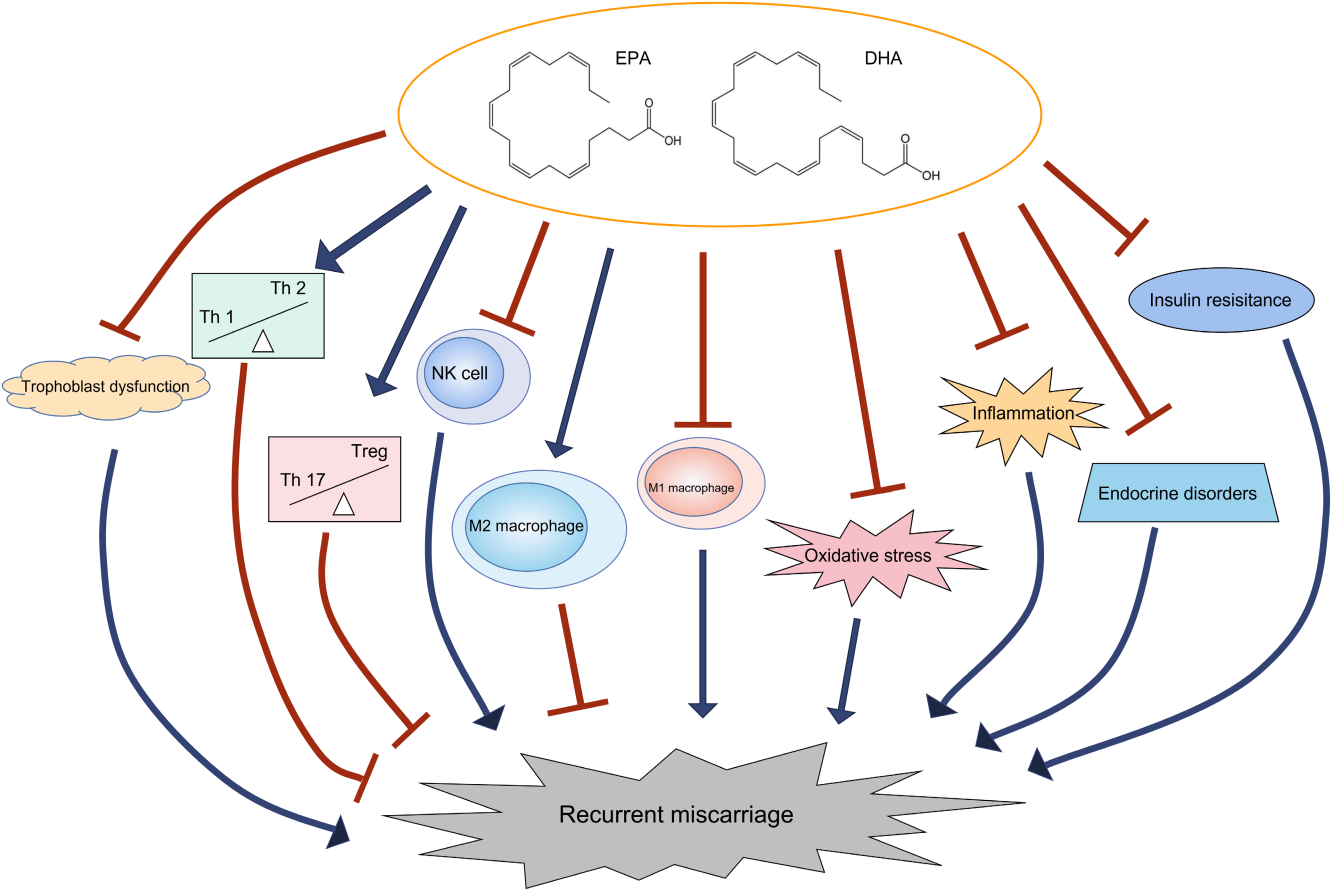
The hypothesized mechanism of omega‐3 fatty acids in treating RM.

Previous studies have implied that trophoblast dysfunction is considered an underlying reason for miscarriage (Knofler et al., 2019). For instance, one study suggests that a low level of insulin‐like growth factor‐binding protein 7 (IGFBP7), a member of the IGFBP family that regulates cell growth, proliferation, and differentiation, is noted in specimens of patients with RM, and knockdown of IGFBP7 inhibits matrix metalloproteinase 2 and Slug (a widely expressed transcriptional regulator belonging to the Snail family of zinc finger transcription factors) levels via the insulin growth factor‐1 receptor (IGF‐1R)‐mediated c‐Jun signaling pathway to reduce trophoblast invasion, thus inducing miscarriage (Wu et al., 2022). Another study proposed that wingless/integrated (WNT) family member 16 (WNT16) promotes the survival and invasion of trophoblasts through the Akt/β‐catenin signaling pathway (Li, Shi, et al., 2022). In addition, it has been reported that long noncoding RNA‐HZ04 (lncRNA‐HZ04) binds with microRNA‐HZ04 (miR‐HZ04) to affect the stability of type 1 inositol 1,4,5‐trisphosphate receptor (IP_3_R_1_), subsequently activating the Ca^2+^‐mediated IP_3_R_1_/phospho‐calmodulin‐dependent protein kinase II (p‐CMKII)/β‐sarcoglycan (SGCB) signaling pathway, thus increasing trophoblast apoptosis. Moreover, psoralen, a phytochemical compound that is traditionally used for treating psoriasis combined with ultraviolet, promotes the viability and invasion ability of trophoblasts and elevates the expression and activity of MMP‐2 and MMP‐9, as well as the nuclear accumulation and translocation of p65, suggesting that psoralen protects miscarriage by activating the nuclear factor‐κB pathway (Qi et al., 2022). Indeed, regulating trophoblast dysfunction and the underlying molecular mechanisms has become a research hotspot in recent years. It could be assumed that this research field may identify potential treatment targets and generate novel treatment options for RM in the near future; however, further validation is still needed (Figure 1).

#### 2.2.2 Genetic polymorphism

In recent years, studies have focused on the genetic risk factors for RM. For instance, Kwon et al. (2022) evaluated 388 patients with RM and 280 controls in Korea and revealed that the rs10515478 C>G polymorphism of the SMAD5 gene and the rs1046875 G>A polymorphism of the fructosamine 3 kinase‐related protein (FN3KRP) gene are associated with a lower risk of RM. A study conducted by Talwar et al. (2022) suggests that the 844INS68 polymorphism of cystathionine beta‐synthase (CBS) combined with the A2756G polymorphism of 5‐methytetrahydrofolate‐homocysteine methyltransferase (MTR) is associated with over 2‐fold increased RM risk. Salimi et al. (2020) presented a meta‐analysis including 31 case–control studies and revealed that 174 G>C and 634 G>C polymorphisms of the interleukin‐6 (IL‐6) gene and 137 G>C polymorphisms of the IL‐18 gene are associated with a higher risk of RM. Another meta‐analysis including 18 case–control studies conducted by Su et al. (2011) indicated that vascular endothelial growth factor (VEGF) 1154 G>A and endothelial nitric oxide synthase (eNOS) Glu298Asp polymorphisms are closely associated with the risk of RM. These studies have highlighted the potential genetic risk factors for RM. However, more studies are encouraged to achieve a deep understanding of RM.

#### 2.2.3 Immunity, oxidative stress, and inflammation in RM

The dysregulation of immunity, existence of oxidative stress, and high level of inflammation are thought to be key parameters in the pathogenesis of RM (Figure 1). Immune cells such as natural killer (NK) and T cells have a great impact on RM. A previous study disclosed a high cytotoxic activity of NK cells in RM patients in the luteal phase, which suggests that NK cell cytotoxic activity is responsible for pregnancy loss in patients with RM (Sokolov et al., 2019). Another study revealed that tumor necrosis factor (TNF)‐α^+^/CD56^+^ NK cells are associated with the risk of pregnancy loss in RM patients (Takeyama et al., 2021). A recent study identified a novel subset of NK cells, CXCR4^+^ CD56^bright^ decidual NK cells, which are insufficiently expressed in RM patients and RM model mice; in addition, adoptive transfer of these cells to NK cell‐deficient mice improves pregnancy outcomes (Tao et al., 2021). Notably, several studies conducted with a mouse model of RM suggested that the modulation of NK cells in abortion‐prone mice is able to promote pregnancy outcomes (Li et al., 2017; Nagamatsu et al., 2018; Rezaei Kahmini et al., 2020; Tanaka et al., 2016). T cells are also critical modulators of immunity. For instance, the adoption of CD4^+^CD25^+^Foxp3^+^ regulatory T (Treg) cells reduces abortion in RM mice (Wang et al., 2019). Meanwhile, it has also been reported that the polarization of T cells toward T‐helper (Th)2 cells promotes pregnancy maintenance in abortion‐prone mice (Li, Sun, et al., 2022). In patients with RM, it is reported that the Th1/Th2 ratio is increased (Kuroda et al., 2021). Another study suggests that the Treg/Th17 ratio is significantly decreased in patients with RM compared to individuals with normal pregnancies (Ji et al., 2019). In terms of macrophages, it is suggested that the promotion of autophagy and cell resistance in decidual macrophages improves pregnancy maintenance in RM model mice (Yang et al., 2022). In addition, a higher level of M1 polarization of macrophages prevents pregnancy loss in RM model mice (Cui et al., 2021). Clinically, apoptosis and efferocytosis are higher in the macrophages of RM patients than in those of normal pregnancies (Sheng et al., 2022). In addition, M1 macrophages are abundant, but M2 macrophages are insufficient in the deciduae of patients with RM compared to those with normal pregnancies (Tsao et al., 2018).

Oxidative stress, caused by the imbalance of peroxidants and antioxidants, participates in various pathological processes, including RM. A previous study suggests that the levels of heat shock protein 70, nitrotyrosine, and lipid peroxidation are all elevated in placental tissues from miscarriages compared with those from normal pregnancies (Hempstock et al., 2003). The high level of oxidative stress hampers placental development, which induces pregnancy loss (Gupta et al., 2007). Clinically, numerous studies have implied that the level of oxidative stress is associated with RM. For instance, total antioxidant capacity is significantly decreased in RM patients, while the level of a DNA damage marker related to oxidative stress, 8‐hydroxydeoxyguanosine, is increased in RM patients (Alrashed et al., 2021). Other markers of oxidative stress, including oxidized glutathione (Ghneim & Alshebly, 2016), malondialdehyde (Al‐Sheikh et al., 2019), and nitric oxide (NO; Raffaelli et al., 2010), are all increased, but the levels of antioxidants, including superoxide dismutase (Ghneim et al., 2016) and glutathione (Ghneim & Alshebly, 2016), are decreased in RM patients.

The high level of inflammation may induce apoptosis in trophoblast cells, which could result in pregnancy loss (Alfian et al., 2022; Yougbare et al., 2017). Meanwhile, the levels of inflammatory cytokines can be modulated by immune cells such as NK cells and Th cells. As mentioned earlier, these immune cells critically modulate RM; thus, inflammatory cytokines also participate in the pathogenesis of RM. On the other hand, inflammation also regulates insulin resistance, obesity, and polycystic ovary syndrome, which are known factors associated with RM (Chakraborty et al., 2013; Sugiura‐Ogasawara, 2015). Indeed, clinical studies have reported the dysregulation of inflammatory cytokines in patients with RM (Lob et al., 2021; Peng et al., 2021; Qian et al., 2018).

## 3 POTENTIAL INVOLVEMENT OF OMEGA‐3 FATTY ACIDS IN RM

Omega‐3 fatty acids are a group of polyunsaturated fatty acids that exert multiple biological functions, such as regulating cell survival, inhibiting inflammation and oxidative stress, modulating immunity, and modifying the endocrine system.

### 3.1 Omega‐3 fatty acids regulate trophoblast dysfunction

Trophoblast dysfunction is critically associated with RM. According to some previous studies, omega‐3 fatty acids exert regulatory effects on trophoblast dysfunction. For instance, DHA (25 μM) attenuated lipopolysaccharide‐induced inflammation in trophoblast cell lines and decreased the preterm delivery of pregnant mice treated with LPS (Chen et al., 2018). A previous study suggested that DHA (12.5–100 μM) promoted the tube length and secretion of vascular endothelial growth factor in a trophoblast cell line in a dose‐dependent manner (Johnsen et al., 2011). Similar findings were also reported in another study (Basak & Duttaroy, 2013). It has also been reported that 100 mM DHA significantly induced oxidative stress in a trophoblast cell line, while 1 and 10 mM DHA markedly decreased oxidative stress; pretreatment with 1 and 10 mM DHA also promoted the survival rate of trophoblasts under oxidative stress induced by H_2_O_2_ treatment (Shoji et al., 2009). These studies suggested the protective effect of DHA on trophoblast dysfunction, while the evidence of EPA on this is relatively lacking.

### 3.2 Omega‐3 fatty acids modulate immunity

Accumulating evidence has shown that omega‐3 fatty acids exert an immunoregulatory effect. One study showed that a fish oil‐enriched diet (4.0 g EPA and 2.9 g DHA per kg diet) increased CD11B^+^CD27^−^ NK cells, CD107a^+^ NK cells, and CCR5^+^ NK cells after inflammation induction in mice (Jensen et al., 2022). In cancers, the activity of NK cells is inhibited (Seliger & Koehl, 2022). Exosomes from an omega‐3 fatty acid (12.5 μM EPA or DHA)‐treated multiple myeloma cell line (L363) were able to restore the activity of NK cells (NK‐92; Moloudizargari et al., 2020). In addition, it has also been reported that a daily omega‐3 fatty acid‐enriched diet (containing 1 g omega‐3 fatty acid) for 6 months reduced NK cell numbers and resulted in lower inflammation in healthy subjects (Mukaro et al., 2008). Although no studies have revealed the effect of omega‐3 fatty acids under RM conditions, it is assumed that they might also restore NK cells to a normal level.

The regulatory effect of omega‐3 fatty acids on T cells has been reported by a number of studies. It has been suggested that an omega‐3 fatty acid‐enriched diet (containing 5% omega‐3 fatty acids) increased CD4^+^Foxp3^+^ and CD4^+^Foxp3^+^CD25^+^ Tregs that suppress inflammation in mice; mechanically, omega‐3 fatty acid reduced Erk and Akt phosphorylation (Camacho‐Munoz et al., 2022). Meanwhile, another study revealed that compared with a high‐fat diet, *ex vivo* coculture of primary mouse CD4^+^ T cells with adipocytes from mice consuming high‐fat plus omega‐3 fatty acid (containing 5.3% kcal from menhaden fish oil) reduced Th1‐related cytokines but increased Th2‐related cytokines and lowered the level of the NOD‐like receptor family pyrin domain containing 3 (NLRP3) inflammasome (Liddle et al., 2021). Moreover, supplementation with ALA, EPH, or DHA (100 μM) in a coculture system of human adipose‐derived stem cells from obese and human mononuclear cells inhibited interleukin (IL)‐17, which is mainly secreted by Th17 cells (Chehimi et al., 2019).

In terms of the effect of omega‐3 fatty acids on macrophages, one study reported that in healthy human‐derived macrophages cultured with a differentiation medium containing omega‐3 fatty acids (20 μM), M1 macrophage differentiation was suppressed, but M2 macrophage differentiation was promoted (Schwager et al., 2022). Another study revealed that omega‐3 fatty acid (25 μM) reduced the production of NO and alleviated the level of inflammatory status in macrophages from type 1 diabetes mellitus mice (Davanso et al., 2021). Similar findings were observed in macrophages from patients with abdominal aortic aneurysm, in which *ex vivo* omega‐3 fatty acids (20 and 80 μM) reduced the production of proinflammatory cytokines in these macrophages (Meital et al., 2019).

Although these studies have indicated the regulation of omega‐3 fatty acids in immunity, it should be considered that the immune condition between RM and the circumstances above (such as inflammation, diabetes mellitus, and abdominal aortic aneurysm) may vary to some extent. Thus, the effect of omega‐3 fatty acids on immunity in patients with RM still needs further verification.

### 3.3 Omega‐3 fatty acids modulate oxidative stress

Several studies have disclosed the effect of omega‐3 fatty acids on oxidative stress. One study reported that consuming a diet enriched with omega‐3 fatty acids (containing 2.68% EPA and 3.17% DHA) for3 months reduced the level of thiobarbituric acid–reactive substances, a lipid peroxidation marker, in police dogs; meanwhile, the activity of glutathione peroxidase (GPx) was elevated compared with dogs consuming a normal diet (Ravic et al., 2022). Another study administered DHA‐enriched fish oil (450 mg/kg body weight twice daily) to stress‐induced brain oxidative stress model mice; the authors disclosed a lower level of serum lipid peroxidation and a higher level of serum total antioxidant capacity (Asari et al., 2022). Meanwhile, in rats fed a high‐fat diet, the addition of omega‐3 fatty acids (50.79 mol% DHA + EPA) reduced lipid peroxidation levels and increased GPx activity (Miralles‐Perez et al., 2021). One previous study explored the effect of omega‐3 fatty acids on oxidative stress under the scenario of cigarette smoke (Wiest et al., 2017). The authors fed the mice a control diet or omega‐3 fatty acid diet (containing 1.8% ALA, 16.0% EPA, and 10.8% DHA) for 8 weeks and then exposed the mice to cigarette smoke for 5 days, 2 h per day. Cigarette smoke‐induced oxidative stress markers were significantly reduced in mice fed an omega‐3 fatty acid diet compared with mice fed a control diet (Wiest et al., 2017). A recent meta‐analysis reviewed 39 trials with 2875 subjects who either consumed omega‐3 fatty acid supplements or placebo (Heshmati et al., 2019). The pooled analysis showed that total antioxidant capacity, GPx, and reduction of malondialdehyde were significant in participants who received omega‐3 fatty acid supplements compared with those who received placebo; however, the changes in NO, glutathione, superoxide dismutase, and catalase activities were not significant between participants receiving different supplements (Heshmati et al., 2019). However, the molecular mechanisms by which omega‐3 fatty acids regulate oxidative stress are still not clear and require further exploration.

### 3.4 Omega‐3 fatty acids modulate the endocrine system

There is some evidence suggesting that omega‐3 fatty acids are able to modulate endocrine function. For instance, a randomized, controlled trial revealed that in patients with polycystic ovary syndrome, omega‐3 fatty acid (2000 mg/day omega‐3 fatty acids) combined with vitamin D (50,000 IU every 2 weeks) reduced the level of testosterone, ameliorated inflammation, improved total antioxidant capacity, and promoted mental health compared with placebo (Jamilian, Samimi, Mirhosseini, Afshar Ebrahimi, Aghadavod, Talaee, et al., 2018). Similar findings have also been reported in other randomized controlled trials (Amini et al., 2018; Rahmani et al., 2017; Sadeghi et al., 2020). Meanwhile, a recent meta‐analysis included 10 observational studies and revealed that omega‐3 fatty dietary intake reduced the risk of endocrine‐related cancers, such as ovarian cancer and endometrial cancer (Hoang et al., 2021). Moreover, one study reported that in rats with hyperthyroidism‐induced hepatic dysfunction, supplementation with omega‐3 fatty acids (3 g/kg/day containing 18% EPA and 12% DHA) plus L‐thyroxine decreased the serum level of triiodo‐l‐thyronine compared with L‐thyroxine alone; the authors also illustrated that the total antioxidant capacity and inflammation level were reduced by omega‐3 fatty acids plus L‐thyroxine compared with L‐thyroxine alone (Gomaa & Abd El‐Aziz, 2016). Considering that the pathogenesis of RM is closely associated with endocrine dysfunction, such as polycystic ovary syndrome, hyperthyroidism, and hypothyroidism. (Amrane & McConnell, 2019), it is likely that omega‐3 fatty acids might modulate RM through endocrine regulation. However, there is no direct evidence illustrating the regulation of omega‐3 fatty acids in the RM‐related endocrine system, which should be explored in the future.

## 4 CLINICAL PERSPECTIVE OF OMEGA‐3 FATTY ACID SUPPLEMENTS IN RM

Until now, some clinical studies have implied the effect of omega‐3 fatty acid supplements in RM. The direct evidence that omega‐3 fatty acids are used to treat RM is quite limited (Table 1). However, there are some studies indicating that omega‐3 fatty acid supplements could modulate inflammation, insulin resistance, thyroid antibodies, and oxidative stress in pregnant women, which, as mentioned above, is deeply associated with RM (Table 2).

**TABLE 1:** Direct evidence from clinical studies indicating the effect of omega‐3 fatty acids on RM.

References	Study design	Participants	Grouping	Major findings
Lazzarin et al. (2009)	Randomized, controlled trial	60 RM patients	Aspirin (100 mg daily) (*N* = 20) Omega‐3 fatty acids (4 g daily) (*N* = 20) Aspirin (100 mg daily) plus omega‐3 fatty acids (4 g daily) (*N* = 20)	Aspirin plus omega‐3 fatty acids group had the highest uterine blood flow index
Carta et al. (2005)	Prospective study	30 RM patients with antiphospholipid syndrome	Aspirin (100 mg daily) (*N* = 15) Fish oil derivate (4 g daily) (*N* = 15)	Live birth rate, gestational age at delivery, fetal birth weight, cesarean sections, and complications were comparable between groups
Rossi and Costa (1993)	Prospective study	22 RM patients	All received EPA and DHA (5.1 g daily, ratio: 1.5)	Of 23 pregnancies, 19 ended at the 37th week producing a baby, 1 was going on at 32nd week, 2 pregnancies ended with cesarean section for preeclampsia at 30th and 35th week, and 1 intrauterine fetal death at the 27th week

Abbreviations: CRP, C‐reactive protein; DHA, docosahexaenoic acid; EPA, eicosapentaenoic acid; RM, recurrent miscarriage.

**TABLE 2:** Indirect evidence from clinical studies indicating the effect of omega‐3 fatty acids on RM.

References	Study design	Participants	Grouping	Major findings
Sley et al. (2020)	Prospective study	725 pregnant women	Omega‐3 fatty acid supplement (dose not clear) (*N* = 165) No omega‐3 fatty acid supplement (*N* = 560)	Omega‐3 fatty acid supplement was associated with 10.2% lower levels of 8‐iso‐PGF2α and 10.3% lower levels of the metabolite
Jamilian, Samimi, Mirhosseini, Afshar Ebrahimi, Aghadavod, Taghizadeh, et al. (2018)	Randomized, controlled trial	40 women with gestational diabetes mellitus	Fish oil capsule containing DHA (180 mg) and EPA (120 mg) twice daily (*N* = 20) Placebo (*N* = 20)	Fish oil supplement reduced mRNA levels of PPAR‐γ, LDLR, IL‐1, and TNF‐α in PBMCs
Kajarabille et al. (2017)	Randomized, controlled trial	110 pregnant women	Control dairy drink (*N* = 54) Fish oil‐enriched (containing 320 mg DHA and 72 mg EPA) dairy drink daily (*N* = 56)	Fish oil supplement increased superoxide dismutase and catalase at delivery and after 2.5 months
Razavi et al. (2017)	Randomized, controlled trial	120 women with gestational diabetes mellitus	Omega‐3 fatty acid (1000 mg twice daily) (*N* = 30) Vitamin D (50,000 IU every 2 weeks) (*N* = 30) Omega‐3 fatty acid (1000 mg twice daily) + vitamin D (50,000 IU every 2 weeks) (*N* = 30) Placebo (*N* = 30)	Omega‐3 fatty acid plus vitamin D reduced high‐sensitivity CRP while increased total antioxidant capacity and glutathione; it also decreased the incidences of newborns' hyperbilirubinemia and hospitalization compared with other treatments
Benvenga et al. (2016)	Prospective study	236 thyroid disease‐free women	Swordfish (equivalent to 6.3 ± 2.1 g DHA + EPA monthly) (*N* = 48) Oily fish (equivalent to 13.2 ± 5.4 g DHA + EPA monthly) (*N* = 52) Swordfish+other fish (equivalent to 6.0 ± 2.8 g DHA + EPA monthly) (*N* = 68) Fish other than swordfish and oily fish (equivalent to 5.1 ± 3.8 g DHA + EPA monthly) (*N* = 68)	Positive rates and serum concentrations of thyroglobulin and thyroperoxidase antibodies were the lowest in oily fish group
Taghizadeh et al. (2016)	Randomized, controlled trial	60 women with gestational diabetes mellitus	Omega‐3 fatty acid (1000 mg daily) plus vitamin E (400 IU daily) (*N* = 30) Placebo (*N* = 30)	Omega‐3 fatty acid plus vitamin E had beneficial effect on fasting plasma glucose, serum insulin concentration, and serum lipids
Jamilian et al. (2016)	Randomized, controlled trial	60 women with gestational diabetes mellitus	Omega‐3 fatty acid (1000 mg daily) + vitamin E (400 IU daily) (*N* = 30) Placebo (*N* = 30)	Omega‐3 fatty acid promoted total antioxidant capacity but did not affect high‐sensitivity CRP; it also decreased hyperbilirubinemia incidence in newborns
Haghiac et al. (2015)	Randomized, controlled trial	49 obese pregnant women	DHA plus EPA (2 g daily) (*N* = 25) Placebo (*N* = 24)	DHA plus EPA treatment reduced plasma CRP and TLR4 in adipose and placental
Samimi et al. (2015)	Randomized, controlled trial	56 women with gestational diabetes mellitus	DHA plus EPA (1000 mg daily) (*N* = 28) Placebo (*N* = 28)	DHA plus EPA resulted in a greater serum insulin change and lower high‐sensitivity CRP, but did not affect fasting plasma glucose and lipid profiles

Abbreviations: CRP, C‐reactive protein; DHA, docosahexaenoic acid; EPA, eicosapentaenoic acid; IL‐1, interleukin‐1; LDLR, low‐density lipoprotein receptor; PBMCs, peripheral blood mononuclear cells; PGF2α, prostaglandin F2α; PPAR‐γ, peroxisome proliferator‐activated receptor gamma; RM, recurrent miscarriage; TLR4, toll‐like receptor 4; TNF‐α, tumor necrosis factor‐α.

### 4.1 Direct evidence

The direct evidence of omega‐3 fatty acids in treating RM is quite limited. In a randomized controlled trial, RM patients with impaired uterine perfusion were assigned to receive daily 100 mg of aspirin, daily 4 g of omega‐3 fatty acids, or daily 100 mg of aspirin plus 4 g of omega‐3 fatty acids. The results revealed that after 2 months of intervention, the uterine artery pulsatility index was increased in all three groups, which suggests that omega‐3 fatty acids may serve as a therapeutic agent for RM due to impaired uterine perfusion (Lazzarin et al., 2009). However, the sample size of this study was not large enough (*N* = 20 in each group), and blinding was not mentioned. Additionally, this study does not investigate the effect of omega‐3 fatty acids on live birth, a critical pregnancy outcome in patients with RM. Another prospective study enrolled 30 RM patients with antiphospholipid syndrome who either received low‐dose aspirin or omega‐3 fatty acids. The findings indicate that the live birth rate was 80% (12/15) in the aspirin group and 73.3% (11/15) in the omega‐3 fatty acid group (*p* > .05). In addition, the gestation duration and fetal birth weight were both similar between groups (Carta et al., 2005). However, the small sample size of this study may result in low statistical power and affect the reliability of the findings. Moreover, a prospective study enrolled 22 RM patients with antiphospholipid syndrome who were treated with fish oil, which was equivalent to 5.1 g of omega‐3 fatty acids. After 3 years of intervention, only one fetal death occurred at the 27th week of gestation. The other 21 pregnancies end with babies of good health (Rossi & Costa, 1993). Again, the sample size of this study is small, and there lacks a control cohort. These studies indicate that omega‐3 fatty acids possess treatment potential for RM. However, the sample sizes of these studies are generally small. Thus, more studies are required to further verify the therapeutic efficacy of omega‐3 fatty acids in patients with RM.

### 4.2 Indirect evidence

There is also some indirect evidence indicating the potential of omega‐3 fatty acid supplements for the treatment of RM. For example, a randomized controlled trial analyzed 49 obese pregnant women who either received placebo (*N* = 24) or daily 2 g of DHA plus EPA (*N* = 25) from weeks 10 to 16 of gestation to term. The results show that plasma C‐reactive protein was reduced in women who received DHA plus EPA. Meanwhile, the adipose tissue and placenta in women who received DHA plus EPA presented lower levels of inflammatory markers (Haghiac et al., 2015). A double‐blinded, randomized, controlled trial revealed that in pregnant women with gestational diabetes mellitus, the administration of 180 mg of EPA and 120 mg of DHA twice a day for 6 weeks increased the mRNA expression of peroxisome proliferator‐activated receptor gamma (PPAR‐γ) and reduced the mRNA expression of low‐density lipoprotein receptor (LDLR), IL‐1, and tumor necrosis factor alpha (TNF‐α) in peripheral blood mononuclear cells, indicating a lower level of insulin resistance (Jamilian Samimi, Mirhosseini, Afshar Ebrahimi, Aghadavod, Taghizadeh, et al., 2018). Similar findings have also been reported by other randomized controlled trials (Samimi et al., 2015; Taghizadeh et al., 2016). In a prospective study, 236 thyroid disease‐free, Caucasian pregnant women were assigned to take‐in swordfish, oily fish, swordfish plus other fish, and fish other than swordfish and oily fish. The serum levels of thyroid antibodies were lowest in women consuming oily fish. Meanwhile, a negative relationship was found between fish consumption and serum levels of thyroid antibodies in women consuming oily fish (Benvenga et al., 2016). A prospective study enrolled 725 pregnant women, among whom 165 women consumed omega‐3 fatty acids in the third trimester. The analysis revealed that the levels of urinary 8‐iso‐prostaglandin F2α, an oxidative stress marker, and its metabolite were lower in women who consumed omega‐3 fatty acids (Sley et al., 2020). This study indicates that omega‐3 fatty acids reduce oxidative stress during pregnancy. Similar findings have also been reported (Jamilian et al., 2017; Kajarabille et al., 2017; Razavi et al., 2017). However, these studies did not directly focus on the effect of omega‐3 fatty acids on pregnancy outcomes in patients with RM. Rather, they could only serve as indirect evidence implying the treatment potential of omega‐3 fatty acids in these patients. In addition, the dosage of omega‐3 fatty acids varies among studies, and the optimal dosage of omega‐3 fatty acids still needs to be investigated. Moreover, most of these studies have a small sample size (fewer than 60 in each group), which would affect the statistical power and reliability of the findings.

## 5 CONCLUSION

RM greatly hampers reproductivity, which is induced or highly correlated with obesity, thyroid dysfunction, polycystic ovary syndrome, antiphospholipid syndrome, dysregulation of immune cells such as NK cells, T cells, and macrophages, oxidative stress, and other factors. In this review, it is clarified that omega‐3 fatty acids may prevent the pathogenesis of RM by modulating the dysregulation of trophoblasts, immune cells, oxidative stress, and endocrine function. In addition, the summarization of clinical evidence suggests that omega‐3 fatty acid supplements may serve as a potential therapeutic agent for RM, either directly improving pregnancy outcomes in patients with RM or indirectly ameliorating inflammation, insulin resistance, oxidative stress, and thyroid dysfunction during pregnancy (Figure 1). However, there is too little evidence directly demonstrating the involvement of omega‐3 fatty acids in the pathogenesis of RM or the efficacy of omega‐3 fatty acids in treating patients with RM. Therefore, future studies should investigate the effect of omega‐3 fatty acids on immunity, oxidative stress, inflammation, and the endocrine system in RM, as well as the underlying molecular mechanisms. More importantly, further clinical studies should be conducted to clarify the therapeutic role of omega‐3 fatty acid supplements in treating RM, thus promoting the outcome of these patients.

## AUTHOR CONTRIBUTIONS

**Fangxiang Mu:** Data curation (equal); funding acquisition (equal); project administration (equal); supervision (equal); writing – review and editing (equal). **Huyan Huo:** Data curation (equal); formal analysis (equal); investigation (equal); methodology (equal); writing – original draft (equal). **Mei Wang:** Investigation (equal); methodology (equal); visualization (equal). **Fang Wang:** Conceptualization (equal); funding acquisition (equal); project administration (equal); resources (equal); supervision (equal); validation (equal); writing – review and editing (equal).

## FUNDING INFORMATION

This study was supported by the Science Foundation of Lanzhou University (Grant No. 071100132) and the Science Foundation of Lanzhou University Second Hospital (Grant No. YJS‐BD‐19), and the Medical Innovation and Development Project of Lanzhou University (Grant No. lzuyxcx‐2022‐137).

## CONFLICT OF INTEREST STATEMENT

The authors declare that they do not have any conflicts of interest.

## ETHICS STATEMENT

This study does not involve any human or animal testing.

## Data Availability

The data that support the findings of this study are available from the corresponding author upon reasonable request.
